# Saddle Pulmonary Embolism Following Total Knee Arthroplasty: A Rare Incidence

**DOI:** 10.7759/cureus.68870

**Published:** 2024-09-07

**Authors:** Wan Muhamad Amir Abdul Halim, Juzaily F Leong, Muhammad Fathi Hayyun, Rizal Abdul Rani, Nor Hamdan Mohamad Yahaya

**Affiliations:** 1 Orthopaedics and Traumatology, University Kebangsaan Malaysia Medical Centre, Kuala Lumpur, MYS

**Keywords:** deep vein thrombosis, pulmonary embolism, saddle pulmonary embolism, total joint arthroplasty, total knee arthroplasty, venous thromboembolism

## Abstract

Pulmonary embolism (PE) is a life-threatening condition that occurs due to the blockage of the pulmonary artery by blood clots. The occurrence of PE after total knee arthroplasty (TKA) is quite rare. Individuals with a history of PE have a high risk of recurrent venous thromboembolism (VTE). We have encountered a case of saddle PE (SPE) following TKA. The patient underwent a TKA due to advanced osteoarthritis. She started to develop respiratory distress after day 2 of surgery. Further investigation showed that she developed SPE. The conclusion from this case is that, while the occurrence is rare, it is critical to identify the risk factors for each patient prior to surgery. Individuals with VTE are at risk of developing recurrent VTE. Those with a previous history of VTE may require long-term anticoagulant medication to prevent a recurrence. Early diagnosis of the risk factor for VTE before the surgical procedure helps assure a positive outcome and prognosis following the procedure. As an additional benefit, it will lower the rates of perioperative morbidity and mortality.

## Introduction

Pulmonary embolism (PE) is a life-threatening condition in which blood clots from leg venous circulation embolize the pulmonary artery, obstructing pulmonary blood flow. Saddle PE (SPE) is a severe type of PE in which a blood clot forms at the bifurcation (split) of the major pulmonary artery, affecting both the right and left pulmonary arteries. This blockage can considerably limit blood flow to the lungs, which can be fatal if not addressed immediately. The term "saddle" refers to the clot's form, which resembles a saddle above the bifurcation point. This condition may disturb gas exchange in the pulmonary circulation and cause respiratory distress. Symptoms may include sudden shortness of breath, chest pain, a high heart rate, and, in severe cases, collapse or shock. Researchers have identified several risk factors associated with this condition, including age over 60, obesity, active cancer, postpartum period, past history of venous thromboembolism (VTE), hormonal replacement therapy, prolonged immobilization, lower limb surgery, and medical conditions such as sickle cell disease, heart disease, hereditary hyeprcoagulable state, acute infection, and inflammatory conditions. The incidence of SPE following total knee arthroplasty (TKA) is very low. A systematic review and meta-analysis reported that the incidence of PE following TKA is 0.01% to 0.27% [1]. Due to the low incidence of PE following TKA, we encountered a case of SPE in our center following TKA and would like to share our experience in managing this case.

## Case presentation

**Figure 1:**
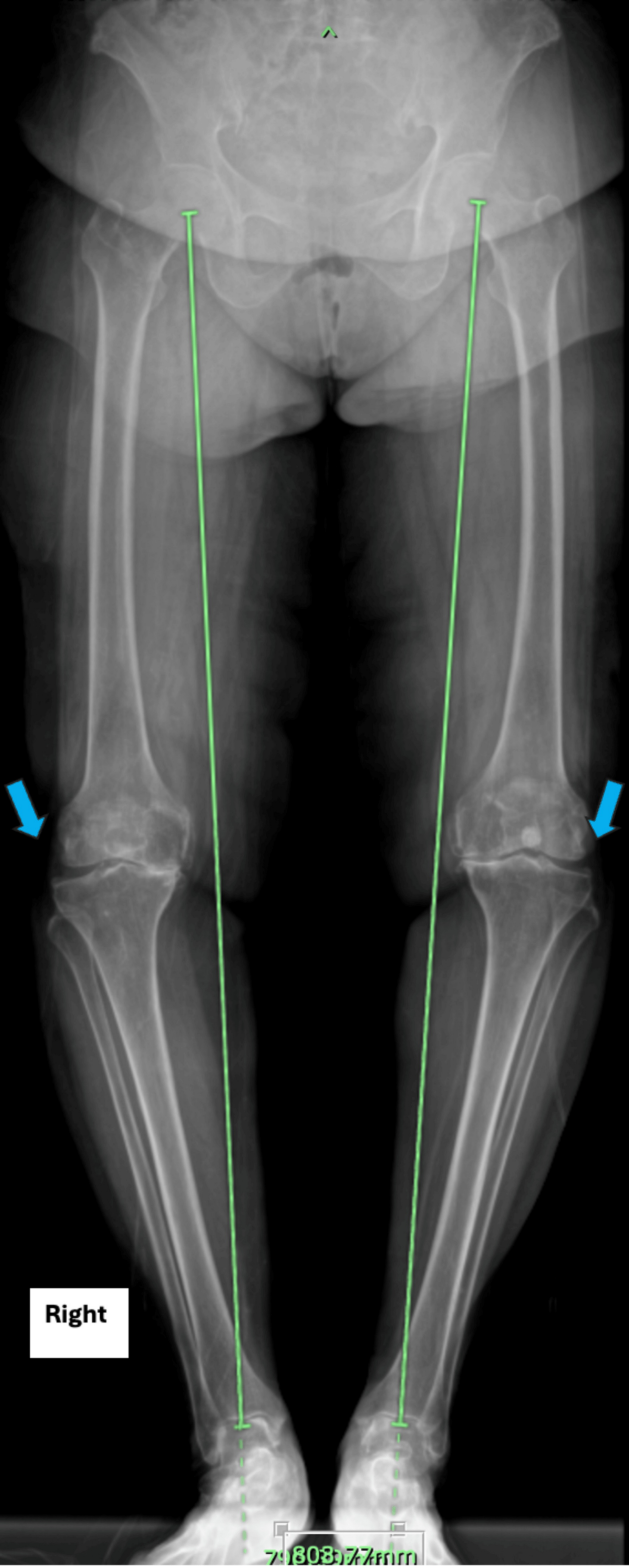
Plain X-ray of the bilateral lower limb showed genu varus deformity with osteoarthritis changes in the bilateral knee.

**Figure 2:**
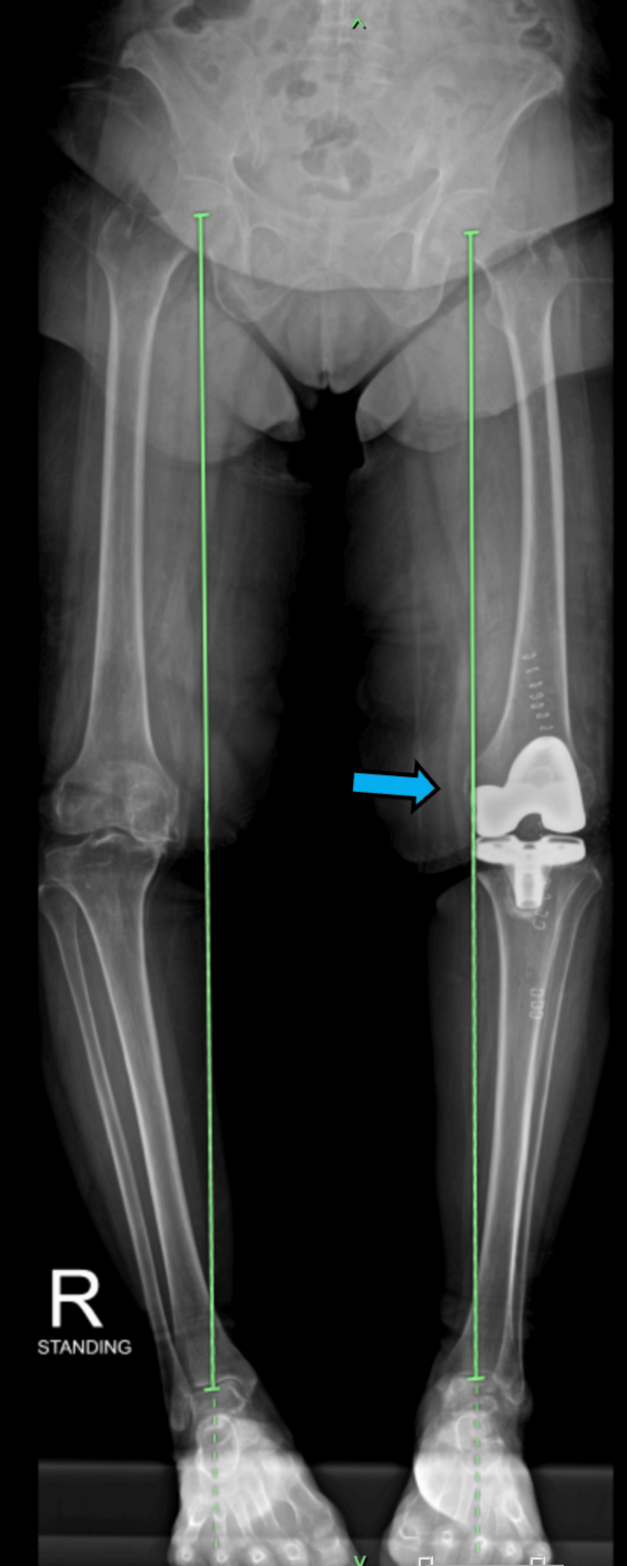
Plain X-ray of the bilateral lower limb showed implant in situ and the mechanical axis of the left lower limb improved after left total knee arthroplasty.

**Figure 3:**
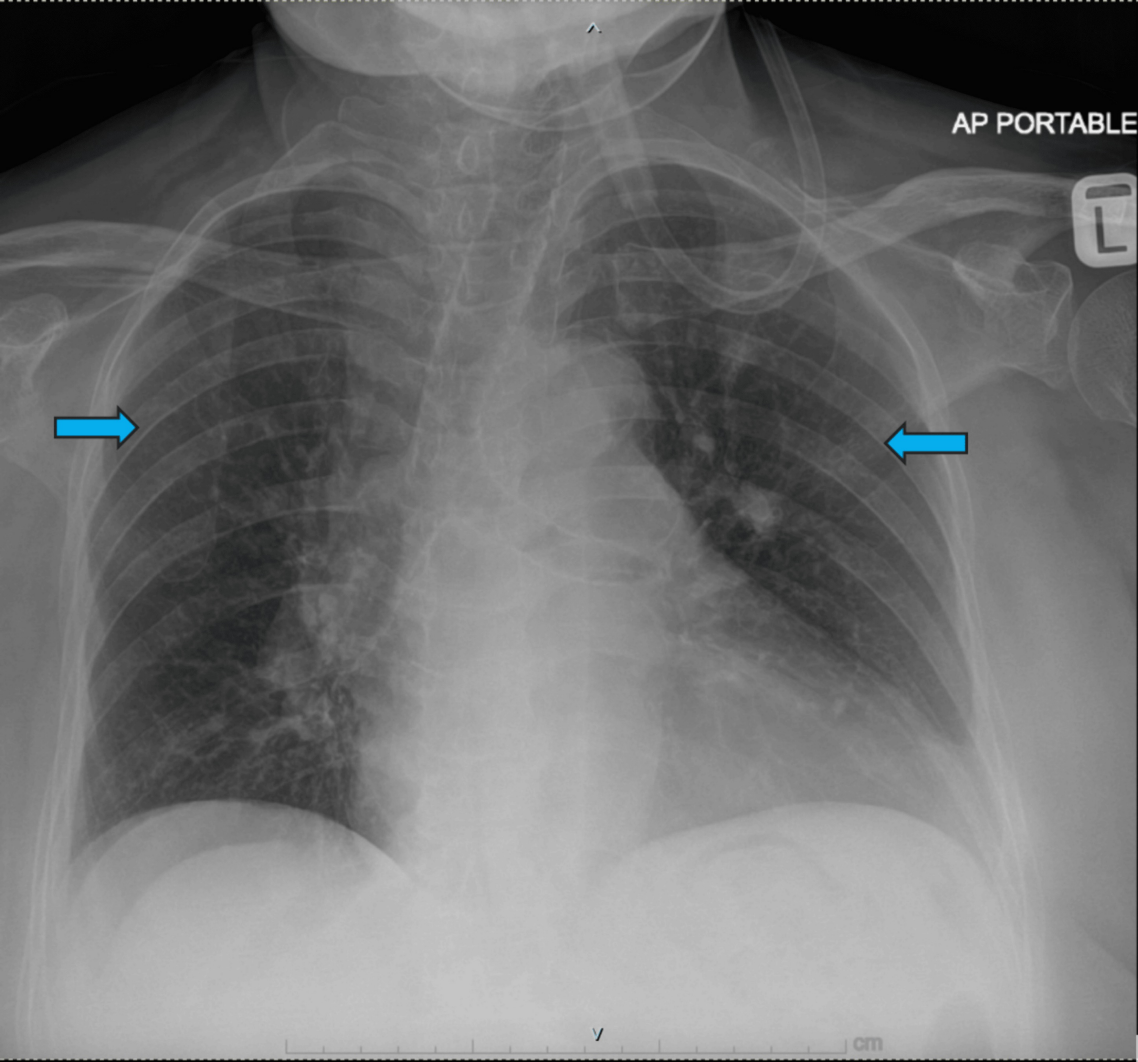
Chest X-ray showed no signs of pneumonia.

A 77-year-old woman was diagnosed with bilateral knee osteoarthritis (Figure 1). She underwent multiple intraarticular knee injections and conservative treatments. After a few years of conservative treatment, she was not satisfied with her disease progression. She experienced functional limitations because she could not stand or walk independently due to severe knee pain. She underwent left TKA (Figure 2) in view of worsening bilateral knee osteoarthritis and failed conservative management. The surgery was performed under general anesthesia, and her vital signs and her condition during surgery were good, without any episodes of desaturation. The patient was started with a calf pump device and thrombo-embolus deterrent (TED) stockings following surgery as a mechanical deep vein thrombosis (DVT) prophylaxis. She was in good health the day after the operation, but on the second day, she began to experience respiratory distress. Her oxygen saturation dropped to 80%, and her arterial blood gas showed type I respiratory failure. Her lung condition on the chest X-ray was good, with no signs of pneumonia (Figure 3).

**Figure 4:**
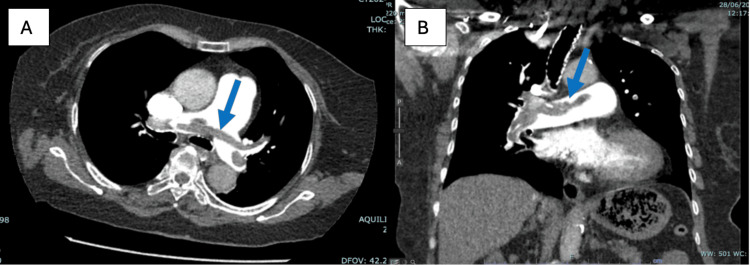
CT pulmonary angiogram (A: axial view and B: coronal view) showed extensive saddle embolism at the bifurcation of the pulmonary trunk (the arrow indicates the pulmonary thrombus).

**Figure 5:**
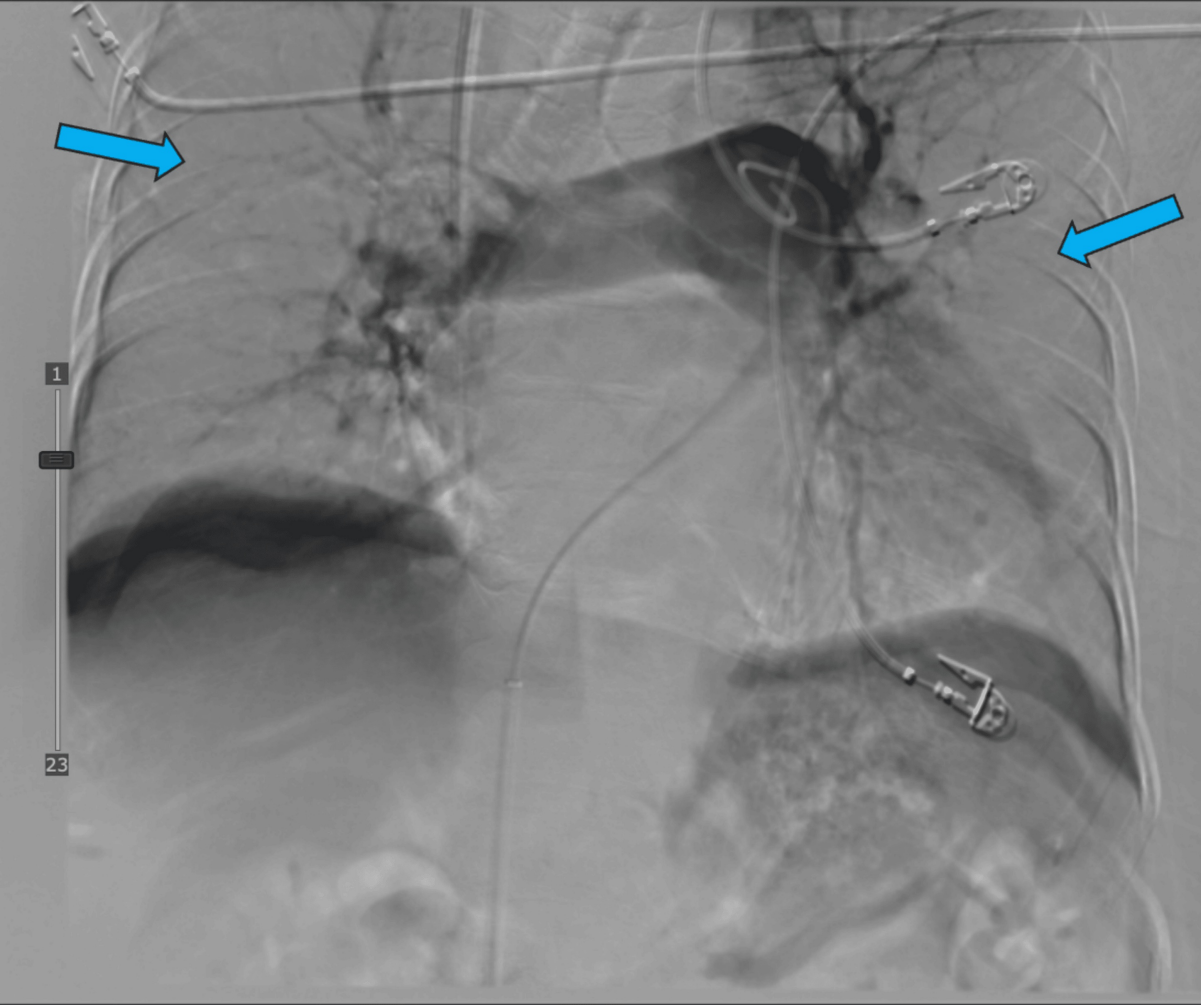
Filling defects (arrow) were seen in the main trunk.

**Figure 6:**
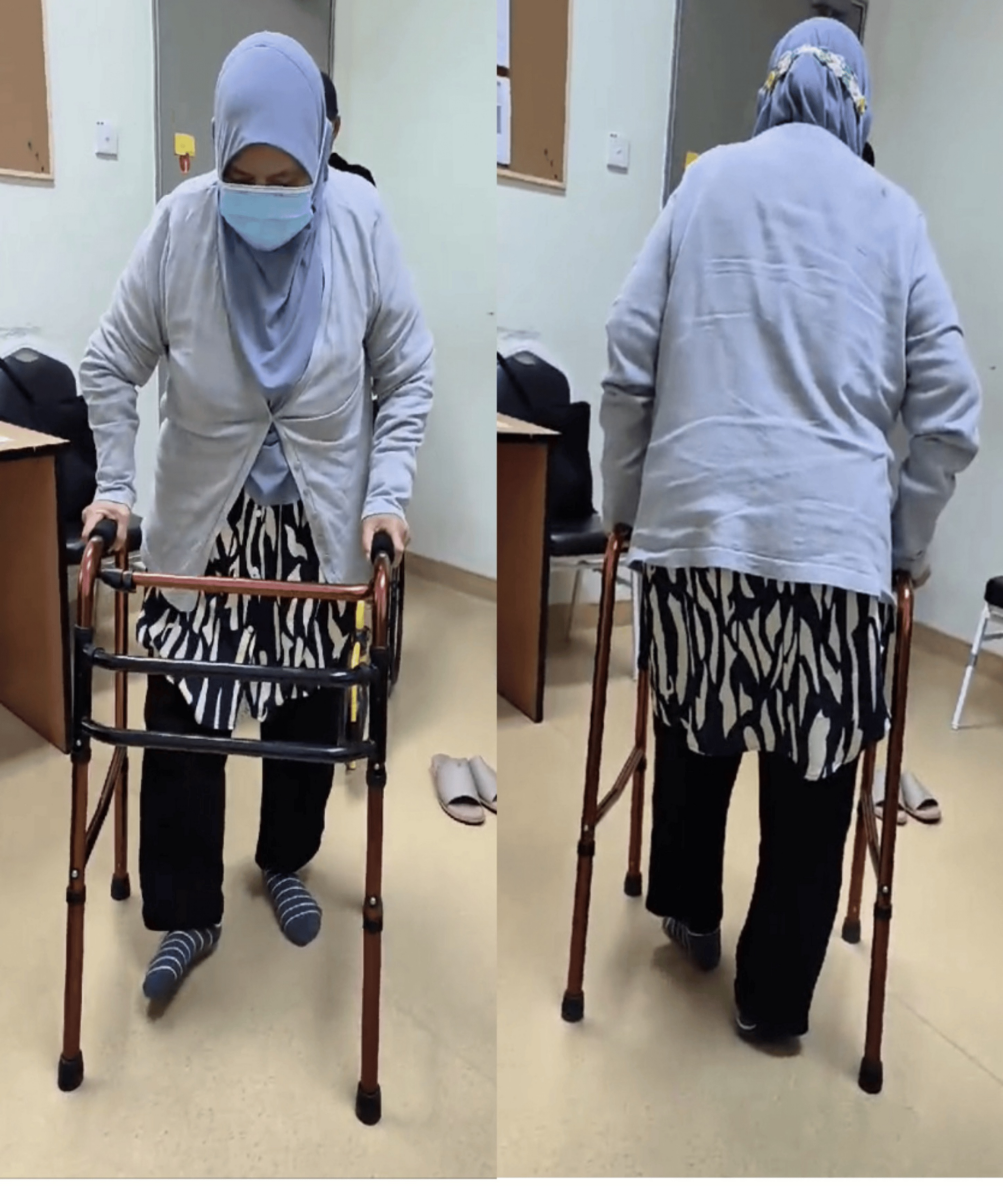
Follow-up after six weeks showed the patient is able to ambulate using a walking frame.

She underwent a computed tomography (CT) scan of the pulmonary angiogram to rule out pulmonary embolism. The CT pulmonary angiogram showed extensive saddle embolism at the bifurcation of the pulmonary trunk, extending to the bilateral main pulmonary arteries and segmental branches (Figure 4). A bilateral pulmonary artery embolism thrombectomy was performed on her by the interventional radiologist. During the thrombectomy procedure, filling defects were seen in the main trunk (Figure 5), bilateral pulmonary arteries, and its branches. The procedure ended uneventfully, and her oxygen saturation improved afterward. Immediately after thrombectomy, she was started on low-molecular-weight heparin for six days, and thereafter, it was changed to dabigatran upon discharge. She was doing much better after surgery than she had been before. We reviewed her condition after six weeks and found that she could walk with the use of a walking frame (Figure 6). 

## Discussion

TKA is a surgical procedure that replaces a diseased or damaged knee joint with an artificial joint (prosthesis). This procedure is typically performed to relieve pain and restore function in patients whose knee joints have been severely damaged by arthritis or injury. Any surgical procedure carries the potential for complications. TKA can cause a variety of problems, including prosthetic joint infection, iatrogenic fracture, vascular damage, and VTE. It was reported in the literature that the incidence of VTE following TKA was 0.32% [2]. VTE refers to a condition where blood clots form in the veins. This typically includes two related conditions, which are DVT and PE. DVT occurs when a blood clot forms in a deep vein, usually in the legs, which can cause pain, swelling, and redness in the affected limb. PE happens when a blood clot dislodges from its site of origin (often in the legs) and travels to the lungs, where it blocks blood flow, which can be life-threatening and requires immediate medical attention. Factors that increase the risk of developing VTE include prolonged immobility (such as after surgery or during long flights), trauma to veins, pregnancy, certain medical conditions (like cancer or clotting disorders), and medications like birth control pills or hormone replacement therapy. 

SPE is a severe type of PE defined by the formation of blood clots near the pulmonary artery bifurcation. The term "saddle" refers to the morphology of the clot, which is positioned like a saddle above the bifurcation point. The incidence of PE after TKA is relatively low. A few studies have found that the incidence of PE after TKA ranges from 0.01% to 0.28% [1-5]. Despite its low occurrence, this illness is life-threatening and can have a significant impact on sufferers. Individuals suffering from PE have a 17% risk of recurrent PE and may require long-term anticoagulant therapy [6,7]. The goal of anticoagulation after acute PE care is to complete the acute episode's treatment and prevent VTE recurrence over time. Clinical trials have evaluated different durations of anticoagulant therapy. The best duration of anticoagulation remains unknown and must be determined on a case-by-case basis. A minimum of three months is often suggested, but a longer duration is required if the PE is unprovoked or if persistent risk factors are present. There are two choices of anticoagulants used after acute PE, which include non-vitamin K antagonist oral anticoagulants (NOAC) and vitamin K antagonists (VKA). NOACs are tiny compounds that directly inhibit one activated coagulation factor, namely, thrombin for dabigatran and factor Xa for apixaban, edoxaban, and rivaroxaban. Meanwhile, the other choice, apart from NOACs, is VKA, which is warfarin. 

Eichinger et al. (2004) reported that patients with symptomatic PE had a fourfold risk of recurrent PE compared to patients with DVT [6]. Recurrent PE is linked to severe clinical outcomes, resulting in death in around 4-9% of patients [6,8]. Given the high risk of recurrent PE, it is crucial to identify the risk factor for PE in order to prevent this from occurring. There are a few risk factors associated with PE following total joint arthroplasty (TJA), which include a history of VTE, hereditary hypercoagulable state, revision TKA, simultaneous bilateral TKA, age more than 70 years old, body mass index (BMI) greater than 30, and female sex [4,9,10]. A systematic review published by Zhang et al. (2015) found that TKA was associated with a higher risk of VTE than total hip arthroplasty (THA), but the reason behind this is still unclear [10].

PE causes patients to spend more time in the hospital and incur higher treatment costs, resulting in an economic burden that is likely to become even more severe in the years to come. It is common practice to recommend a postoperative anticoagulation regimen to patients who are undergoing TJA. The routine use of aspirin for prophylaxis against VTE following TJA has increased in popularity nowadays in comparison to warfarin, enoxaparin, and rivaroxban, which are trending down [11]. In a recent meta-analysis of 13 randomized controlled trials involving 6,060 patients receiving TKA and THA, there was no difference in the risk of VTE between aspirin and other anticoagulants [12]. Currently, there is no specific guideline for VTE prophylaxis following TJA. This is done with the intention of reducing the risk of VTE. However, the therapeutic challenge of VTE prevention lies in striking a balance between the postoperative thrombotic risk and the associated problems of anticoagulation, such as bleeding, hematoma formation, and infection.

## Conclusions

TKA is a surgical technique that involves replacing a diseased or damaged knee joint with an artificial joint (prosthesis). This surgery is often used to reduce pain and restore function in individuals with severely damaged knee joints due to arthritis or injury. Any surgical procedure has the potential for problems. One of the most common complications of surgery is PE. SPE after TKA is uncommon compared to non-SPE. Despite the low incidence of SPE following TKA, it is critical to identify risk factors for each patient before surgery. Identifying risk factors for VTE before surgery improves outcomes and prognosis. In addition, it will lower perioperative mortality and morbidity. Those with a history of VTE may need long-term anticoagulant medication to avoid recurrence.
